# A novel assisted reduction method in extra-articular fractures of the distal tibia treated with intramedullary nail

**DOI:** 10.3389/fmed.2024.1444434

**Published:** 2024-07-26

**Authors:** Jun Liu, Simin Dai, Lijin Liu, Hailin Kuang, Liang Yan, Qiangqiang Cai, Zongzuan Shao, Wenbo Wei, Zhihai Min, Wubing Tang

**Affiliations:** ^1^Department of Orthopaedic Surgery, The Third Hospital of Nanchang, Nanchang People's Hospital, Nanchang, Jiangxi, China; ^2^Department of Emergency, The Second Affiliated Hospital of Nanchang University, Nanchang, Jiangxi, China

**Keywords:** distal tibial fracture, intramedullary nails, Kirschner wires, Poller screws, blocking screws, fracture fixation

## Abstract

**Background:**

To explore the clinical efficacy and safety of Kirschner wires (KWs) as a blocking screw technique for extra-articular fractures of the distal tibia treated with intramedullary nails (IMNs).

**Methods:**

Fifty-three patients were treated with KW-assisted IMN for extra-articular fractures of the distal tibia via the blocking screw technique or Poller screw (PS) technique. The operation time, number of fluoroscopies, number of blocking screws used, blood loss and time to union were compared between the two groups. Additionally, the functional outcomes of the two groups were compared using range of motion (ROM), visual analog scale (VAS), American Orthopedic Foot and Ankle Society (AOFAS), and Lysholm scores.

**Results:**

Compared with those in the PS group, the operation time in the KW group was significantly shorter, and the number of fluoroscopy procedures and amount of blood loss during KW surgery were also significantly lower (*p* = 0.014, 0.001, and 0.036, respectively). Regarding the functional outcomes, there were no significant differences in the ROM, VAS score, AOFAS score or Lysholm score between the two groups (*p* > 0.05).

**Conclusion:**

In the treatment of extra-articular fractures of the distal tibia with nails, the use of KW as a blocking screw technique is safe and reliable.

## Background

Tibial fracture is one of the most common lower limb fractures and is often caused by high-energy injuries such as traffic accidents, falls, direct attacks, and sports injuries (1). Treatment options can be roughly divided into conservative treatment and surgical treatment. In general, operative fixation is recommended for patients with open fractures, segmental/severely comminuted fractures or closed injuries in which fracture reduction and tibial alignment cannot be effectively maintained (2). The objective of surgical treatment is to correct the angulation, rotation and shortening deformity of the fracture site, achieve bone union of the fracture site, and restore lower limb alignment (3). There are various surgical methods for treating extra-articular fractures of the distal tibia, such as open reduction internal fixation, which requires extensive soft tissue dissection and often leads to postoperative complications such as nonunion and infection (4). In recent years, with the rapid development of minimally invasive techniques, minimally invasive plate osteosynthesis (MIPO) and intramedullary nail (IMN) fixation have become two prevalent surgical methods for extra-articular distal tibia fractures (5). However, a study has shown that treatment of distal tibia extra-articular fractures, MIPO compared with IMN, is associated with a greater risk of incision complications and a longer time to union (6). Currently, IMN is the standard treatment for unstable tibial shaft fractures (7–9).

However, for fractures of the distal tibia, due to the presence of the epiphysis, where the bone marrow cavity is relatively wide, lateral displacement and/or angulation deformity of the fracture site and significant axial instability of the fracture site often occur during the insertion of the IMN (10); when this occurs, the conventional course of action is to artificially narrow the diameter of the medullary cavity of the epiphyseal site by placing blocking screws (Poller screws—PS) (11) and creating lateral pushing force to correct the alignment of the fracture site and maintain the stability of the fracture site (12–14). However, it should be noted that in the process of blocking screw implantation, the steps are complicated, and complications such as bone splitting and blocking screw breakage may occur (14, 15). To the best of our knowledge, no specific studies have reported the application of the KW blocking technique in treating extra-articular fractures of the distal tibia with IMN.

The aim of this study was to assess the clinical efficacy and safety of the KW blocking technique in extra-articular fractures of the distal tibia treated with a nail.

## Patients and methods

### Patients

**Figure 1:**
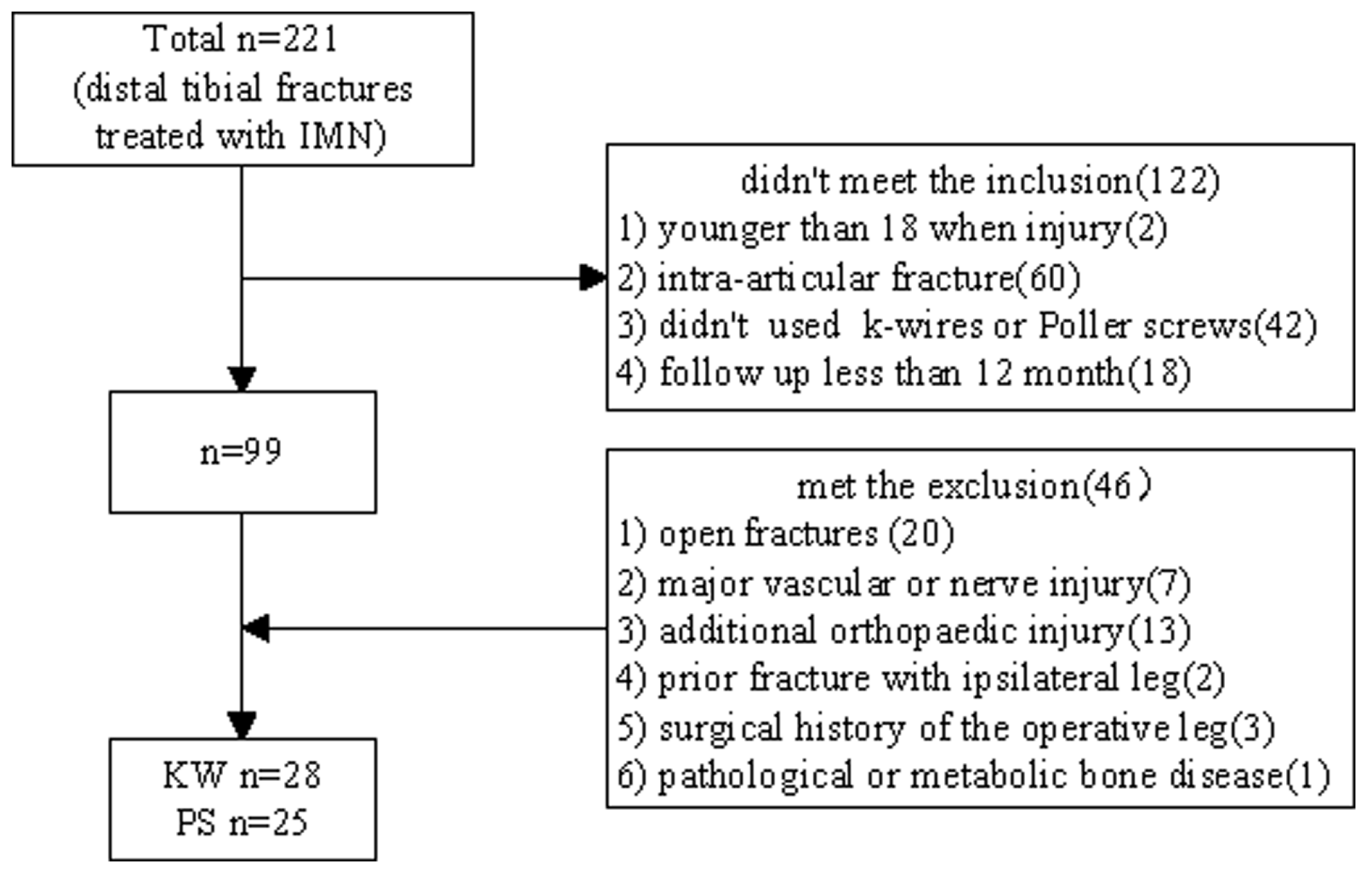
The flowchart for patient selection.

Patients treated with IMN due to distal tibial fracture in our hospital were selected from January 2018 to March 2021. The inclusion criteria were (1) Age > 18 years at the time of injury. (2) Extra-articular distal tibia fracture (AO/OTA: 43. A1-A3). (3) Use KW as temporary blocking or PS technique to assist fracture site reduction during operation. (4) at least 12 months of follow-up. The exclusion criteria were (1) Open fractures. (2) Operative leg with major vascular or nerve injury. (3) Additional orthopedic injury (other than ipsilateral fibula). (4) Prior fracture with the ipsilateral leg. (5) Surgical history of the operative leg. (6) Have pathological or metabolic bone disease (Figure 1).

This study was conducted retrospectively and approved by the Ethics Committee of our hospital. All patients or their authorized relatives signed an informed consent document before receiving a clinical evaluation. The KW group was defined as patients with intraoperative KW-assisted reduction, and the PS group was defined as patients with intraoperative PS-assisted reduction. For the patients included in this study, surgery was performed after the swelling of the injured site went down. The average waiting time for surgery was 4.2 ± 1.4 (range, 2–8) days in the KW group and 4.7 ± 1.6 (range, 2–8) days in the PS group, respectively. All patients in the two groups were followed up for at least 12 months.

## Methods

All patients received intravenous antibiotics of 1 g cefazolin sodium 30 min prior to surgery to prevent infection, and all operations were completed by the same surgical team composed of one senior surgeon and two assistants. After adequate anesthesia, the patients were placed in the supine position. If there was a distal fibula fracture, it was treated with an open reduction plate and screw internal fixation, and then the distal tibia fracture was treated. Conservative treatment was performed for fractures of the middle and upper thirds of the fibula.

**Figure 2:**
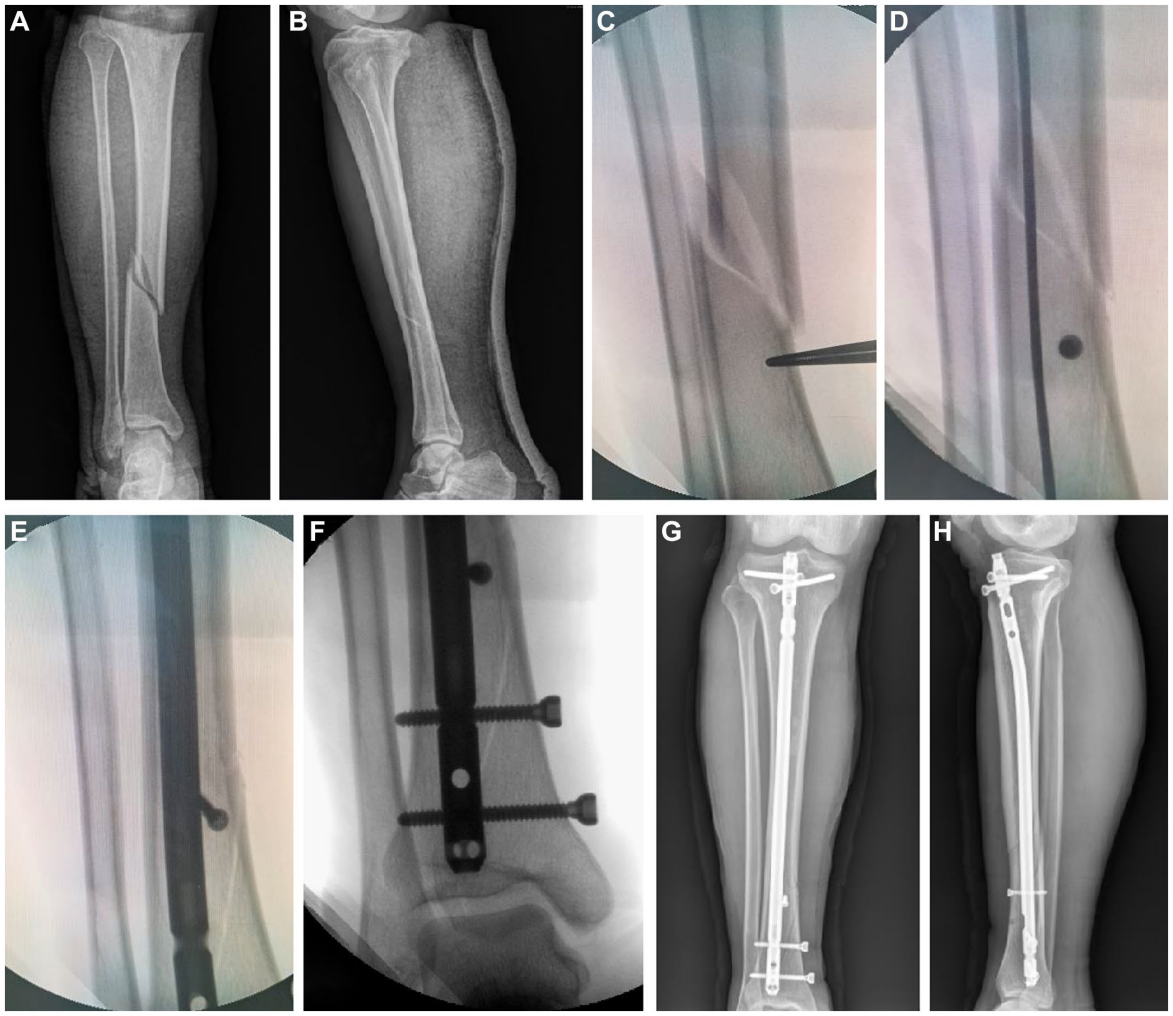
Kirschner wires blocking technique in a 49-year-old female patient treated with nailing. **(A,B)** Pre-operative anteroposterior and lateral view. **(C)** A Kirschner wire was placed for temporary blocking. **(D)** With the assistance of Kirschner wire, the nail was inserted accurately. **(E)** Final nail position, distal locking. **(F)** Kirschner wire was removed at the end of the procedure. **(G,H)** Post-operative anteroposterior and lateral view.

**Figure 3:**
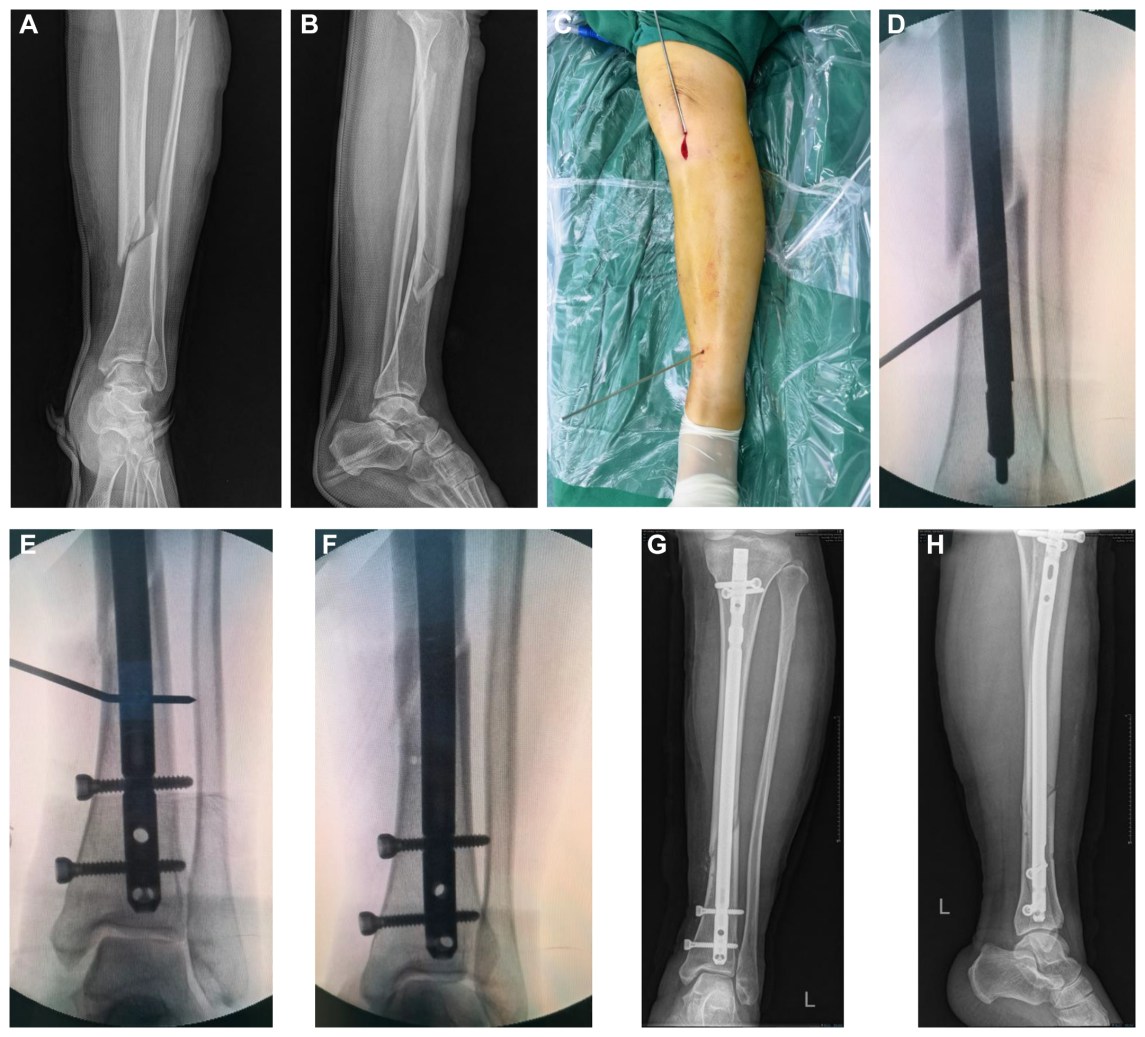
Poller screws technique in a 48-year-old male patient treated with nailing. **(A,B)** Pre-operative anteroposterior and lateral view. **(C)** Location. **(D)** A Poller screws was placed and guidewire inserted. **(E)** Nail inserted. **(F)** Distal locking. **(G,H)** Post-operative anteroposterior and lateral view.

All patients underwent the infrapatellar approach, and the operation before the insertion of the main nail was described by Lu et al. (16). If the IMN (Expert Tibial Nail) was not in the center of the distal tibial medullary cavity after insertion or if the patients had an angular deformity greater than 5° on the coronal plane or 10° on the sagittal plane or obvious lateral displacement at the fracture site, KW, as a temporary blocking or PS technique, was applied to adjust the distal position of the IMN or correct the angular deformity and obvious lateral displacement. For patients in the KW group, the IMN was first withdrawn, and then a KW with a diameter of 2.5, 3 or 3.5 mm or more was inserted percutaneously at the distal fractures of the tibia, the concave side of the angular deformity, or the acute angle side of the connection between the fracture line and the longitudinal axis of the diaphysis, 1 cm from the fracture line, 0.5 cm in the center of the medullary cavity; finally, the IMN was reinserted. At this time, if the position of the IMN is satisfactory and the angular deformity and lateral displacement of the fracture site are corrected, distal and proximal IMN locking screws are inserted successively, the KW is removed, and the ankle joint is moved to check the stability of the fracture site. Otherwise, the position of the KW was adjusted, and the above steps were repeated. Multiple KWs were placed if necessary until the position of the IMN and the alignment of the fracture site were corrected (Figure 2). For the PS group, the methods were described by Krettek et al. (11). First, the IMN was removed, and then suitable blocking screws were inserted percutaneously using the same method used for the KW group; similarly, multiple PSs were placed if necessary until the position of the IMN and the alignment of the fracture site were corrected (Figure 3). All patients received 1 g of cefazolin sodium intravenously again within 24 h after surgery.

### Rehabilitation protocol

On the first day after surgery, the affected limb began to exercise, such as ankle pump movement and quadriceps isometric contraction. Both active and passive exercises of knee motion were started on the first day after surgery, and it was recommended to flex the knee more than 90° at two weeks after surgery. Partial weight-bearing can be started four weeks after surgery, 50% weight-bearing can be carried eight weeks after surgery, and full weight-bearing can be carried three months after surgery generally.

### Clinical assessment

Before discharge, we informed the patients that outpatient follow-up was required 1, 3, 6 and 12 months after the surgery. At each follow-up, radiographs were acquired to evaluate reduction quality and bone union. Data were obtained through the following items in the follow-up of 12 months after the operation: Visual Analog Scale (VAS, 0 = excellent, 10 = Extreme Pain) (17) was used to evaluate postoperative pain relief, and range of motion (ROM) of knee joint, the American Orthopedic Foot and Ankle Society (AOFAS) (18) and Lysholm (19) scores used to assess functional outcomes of the affected limb.

### Statistical analysis

Statistical analysis was performed using SPSS 25.0 (IBM Corp., United States). Continuous variables were expressed as mean ± standard deviation (range), and classification variables were expressed as n (%). Kolmogorov–Smirnov was used to test whether the data were following the normal distribution, in which age and operation time were following the normal distribution data, and the variance was homogeneity, so two independent samples t-test was used. The Mann–Whitney U-test with two independent samples was used for the variables such as the times of fluoroscopy, the amount of blood loss and ROM, which did not conform to the normal distribution or variance. Categorical variables such as gender, cause of injury, and fracture type were tested by the Chi-Square or Fisher’s exact test. *p* < 0.05 was considered statistically significant.

## Results

### General data

Fifty three patients were included, including 28 patients in the KW group (12 males and 16 females), with an average age of 39.2 ± 15.3 (range, 19–68) years. In the PS group, there were 25 patients (16 males and 9 females), mean age 43.3 ± 15.2 (range, 19–70) years. There were no statistically significant differences between the two groups in age (*p* = 0.333), gender (*p* = 0.170), injured left or right legs (*p* = 0.785), ipsilateral fibula accompanying fracture (*p* = 0.894), time from injury to surgery (*p* = 0.199), injury causes (*p* = 0.871) and fracture types (*p* = 0.801). In addition, a total of 28 patients in the KW group were finally followed up, with an average follow-up time of 19.0 ± 6.0 (range, 12–36) months. A total of 25 patients in the PS group were finally followed up, with an average follow-up time of 21.6 ± 5.9 (range, 12–33) months. There was no significant difference in the average follow-up time between the two groups (*p* = 0.063) (Table 1).

**Table 1:** Patient demographics.

	KW	PS	*p*-value
Age	39.2 ± 15.3 (19–68)	43.3 ± 15.2 (19–70)	0.333^a^
Sex, no. (%)			0.170^c^
Male	12(42.9)	16(64)	
Female	16(57.1)	9(36)	
Side, no. (%)			0.785^c^
Left	14(50)	11(44)	
Right	14(50)	14(56)	
Time to surgery (day)	4.2 ± 1.4 (2–8)	4.7 ± 1.6 (2–8)	0.199^b^
Cause of injury,			
No. (%)			0.871^d^
Traffic	17(60.7)	18(72)	
Fall	6 (21.4)	5(20)	
Sport	4(14.3)	2(8)	
Other	1(3.6)	0(0)	
Ipsilateral fibula fracture,			
No. (%)			0.894^d^
Proximal	6(21.4)	7(28)	
Middle	4(14.3)	4(16)	
Distal	8(28.6)	5(20)	
None	10(35.7)	9(36)	
AO/OTA classification,			
No. (%)			0.801^c^
43-A1	15(53.6)	16(64)	
43-A2	8(28.6)	5(20)	
43-A3	5(17.9)	4(16)	
Follow-up (month)	19.0 ± 6.0 (12–36)	21.6 ± 5.9 (12–33)	0.063^b^

^a^*t*-test, ^b^Mann-Whitney U-test, ^c^Chi-squared test. ^d^Fisher’s exact test. Values are expressed as n (%) or the mean ± standard deviation. KW, Kirschner wires; PS, Poller screws.

### Intraoperative comparison

The mean operation time of the KW group was significantly shorter than that of the PS group [62.9 ± 10.6 (range, 48–90) min vs. 71.8 ± 14.8 (range, 55–120) min, *p* = 0.014]. The average times of fluoroscopy of KW were significantly less than that of the PS group [(14.8 ± 1.5 (range, 13–20) vs. 17.8 ± 1.5 (range, 16–22), *p* < 0.001)]. However, there was no significant difference in the numbers of KW and PS [(1.3 ± 0.5 (range, 1–3) vs. 1.2 ± 0.4 (range, 1–2), *p* = 0.288)]. Finally, the blood loss in the KW group was significantly less than that in the PS group [(171.8 ± 55.9 (range, 100–350) mL vs. 218.8 ± 82.0 (range, 100–400) mL, *p* = 0.036)] (Table 2).

**Table 2:** Intraoperative comparison.

	KW	PS	*p*-value
Operation time (min)	62.9 ± 10.6 (48–90)	71.8 ± 14.8 (55–120)	0.014^a^
Fluoroscopy times	14.8 ± 1.5 (13–20)	17.8 ± 1.5 (16–22)	< 0.001^b^
Number of blocking screws	1.3 ± 0.5 (1–3)	1.2 ± 0.4 (1–2)	0.288^b^
Blood loss (mL)	171.8 ± 55.9 (100–350)	218.8 ± 82.0 (100–400)	0.036^b^

^a^*t*-test, ^b^Mann-Whitney U-test, ^c^Chi-squared test. ^d^Fisher’s exact test. Values are expressed as n (%) or the mean ± standard deviation. KW, Kirschner wires; PS, Poller screws.

### Functional outcomes

The AOFAS scores of the KW group and PS group were 93.9 ± 6.2 (range, 74–100, excellent) and 90.0 ± 10.5 (range, 55–100, excellent), respectively (*p* = 0.182). The Lysholm score of the KW group and PS group was 94.6 ± 5.1 (range, 81–100, good) and 91.4 ± 9.8 (range, 60–100, good), respectively (*p* = 0.200). The two groups had no significant difference in AOFAS and Lysholm scores. For VAS and ROM, there was no significant difference between the KW group and PS group [(1.3 ± 1.0 (range, 0–3) vs. 1.2 ± 1.1 (range, 0–3) *p* = 0.711)] and [(0°/141.6° ± 4.7° (range, 130°–145°) vs. 0°/137.8° ± 9.0° (range, 110°–145°)] *p* = 0.114). Finally, the time to union of the KW group was 12.5 ± 1.5 (range, 10–15) weeks, while that of the PS group was 13.6 ± 2.6 (range, 10–22) weeks, the difference was not statistically significant (*p* = 0.108) (Table 3).

**Table 3:** Clinical and functional outcomes of the two groups.

	KW	PS	*p*-value
Fracture healing (weeks)	12.5 ± 1.5 (10–15)	13.6 ± 2.6 (10–22)	0.108^b^
ROM°(extension/flexion)	0/141.6 ± 4.7 (130–145)	0/137.8 ± 9.0 (110–145)	0.114^b^
VAS	1.3 ± 1.0 (0–3)	1.2 ± 1.1 (0–3)	0.711^b^
AOFAS	93.9 ± 6.2 (74–100)	90.0 ± 10.5 (55–100)	0.182^b^
Lysholm	94.6 ± 5.1 (81–100)	91.4 ± 9.8 (60–100)	0.200^b^

^a^*t*-test, ^b^Mann-Whitney U-test, ^c^Chi-squared test. ^d^Fisher’s exact test. Values are expressed as n (%) or the mean ± standard deviation. KW, Kirschner wires; PS, Poller screws; ROM, range of motion; VAS, visual analog scale; AOFAS, American Orthopaedic Foot and Ankle Society; Lysholm, Lysholm Knee Function Score.

### Complications

In the KW group, one patient developed a superficial soft tissue infection at the distal locking screws of the IMN, which was cured after daily dressing change and appropriate antibiotic treatment. In the PS group, one case of new fracture and one case of screw breakage. The other patient had nonunion (Table 4).

**Table 4:** Complications.

	KW	PS	*p*-value
During operation, no. (%)			–
New fracture	None	1(4)	
Screw breakage	None	1(4)	
No	28(100)	23(92)	
Postoperative, no. (%)			–
Infection	1(3.6)	None	–
Delayed union	None	None	
Nonunion	None	1(4)	
No	27(96.4)	24(96)	

KW, Kirschner wires; PS, Poller screws.

## Discussion

Due to the unique advantages of IMN in the treatment of long shaft fractures of limbs, such as avoiding the dissection of soft tissues around the fracture site and the destruction of the periosteum by closed reduction and protecting the blood supply of the fracture site to a great extent, it is highly conducive to promoting bone union and reducing the incidence of infection (7, 20, 21). In addition, several small incisions can be made in IMNs through minimally invasive methods; generally, the maximum incision length is less than 4 cm, and IMNs are widely prevalent among patients (22, 23).

However, in the treatment of distal tibial fractures with IMN, the blocking screw technique is often used by placing blocking screws at an appropriate position at the fracture site to correct the malalignment of the fracture site and the distal position of the nail (24); then, three-point fixation occurs at the isthmus of the long bone, the screws, and the anchorage point at the tip of the nail (25, 26).

However, in clinical practice, the placement of blocking screws requires drilling, length measurement and screw insertion, which are cumbersome. When adjusting the position of the blocking screws, the need for repeated fluoroscopy is inevitable, which increases the radiation exposure of patients and prolongs the operation time. The KW blocking technique is derived from blocking screw logic (27).

In this study, we compared the clinical efficacy and safety of the KW blocking technique with those of the Poller screw technique for the treatment of extra-articular fractures of the distal tibia with nails. The average operation time of the KW group was significantly shorter than that of the PS group, and the amount of blood loss and number of fluoroscopy procedures were significantly lower. However, the functional outcomes of patients in the two groups were similar, preliminarily demonstrating the safety and reliability of the KW technique for temporary blocking. In addition, no obvious malunion or hardware failure occurred in the two study groups. The possible reasons for this observation are as follows. First, after successively inserting the distal and proximal IMN locking screws, the stability of the fracture site was routinely examined by moving the ankle joint. If the stability was insufficient, the number of distal locking screws of the IMN or blocking screws was increased simultaneously or separately (patients in the KW group were assigned to the PS group if they were eventually treated with blocking screws due to poor stability) to ensure that the stability of the fracture site was reliable. However, in clinical practice, such cases are rare. Second, at discharge, we routinely informed patients that they should undergo outpatient review before full weight bearing of the affected leg, which is allowed when an adequate bridging callus is visible on radiographs. Third, all surgeries were performed by a senior doctor in our hospital, and the quality of surgery was guaranteed.

According to our study, the KW blocking technique has the following advantages compared with the blocking screw technique. First, the KW placement procedure is simple and can reduce the operation time and radiation exposure for patients (28). Second, after the distal and proximal locking screws of the IMN were locked successfully, the KW could be removed to avoid the potential risk of skin and subcutaneous soft tissue irritation caused by the indwelling of the blocking screws, and no secondary surgical removal was needed. Third, KW has excellent toughness, and even if it experiences bending deformation during operation, it is difficult to break. Fourth, KW has different diameters, which can be selected according to the specific situation. For example, when the fracture line is closer to the metaphysis, the medullary cavity diameter is larger, or there is significant angular deformity or lateral displacement at the fracture site, we would choose larger KWs, such as 3 mm or 3.5 mm, or even larger. Otherwise, we will choose KWs with a diameter of 2.0 or 2.5 mm. KW is a conventional standby instrument in the orthopedic operating room that can be easily obtained. In addition, with the advent of the Expert Tibia Nail (ETN) (22), in the proximal part, there are five locking options in four planes, and at the distal site, there are four locking options in three planes, with the most distal hole situated 5 mm proximal to the nail tip, which allows the surgeon to achieve improved fixation and angle stable locking. Hence, three-point fixation of the fracture site can be achieved without the need for blocking screws to maintain stability between the fracture sites until bone union is achieved.

This study has several limitations. First, this study was conducted retrospectively, and high-quality randomized controlled trials are needed. Second, the relatively small sample size of this study may limit the generalizability of the results, and larger sample size studies may be needed in the future to confirm the findings. Third, the study only explored the KW blocking technique in extra-articular fractures of the distal tibia; when this technique is applied to other parts of the limb long bone, its functional outcomes need to be further explored.

## Conclusion

The KW blocking technique is safe and reliable for treating extra-articular fractures of the distal tibia with IMN. Compared with PS, the KW blocking technique not only significantly shortened the operation time but also significantly reduced the amount of blood loss and number of fluoroscopy procedures.

## Data availability statement

The raw data supporting the conclusions of this article will be made available by the authors, without undue reservation.

## Ethics statement

The studies involving humans were approved by the Medical Research Ethics Committee of Nanchang Third Hospital. The studies were conducted in accordance with the local legislation and institutional requirements. The participants provided their written informed consent to participate in this study.

## Author contributions

JL: Writing – original draft, Data curation. SD: Data curation, Writing – review & editing. LL: Data curation, Writing – review & editing. HK: Investigation, Writing – review & editing. LY: Formal analysis, Writing – review & editing. QC: Formal analysis, Writing – review & editing. ZS: Formal analysis, Writing – review & editing. WW: Methodology, Writing – review & editing. ZM: Methodology, Writing – review & editing. WT: Supervision, Validation, Visualization, Writing – review & editing.
